# Successful off-pump resection of apical cardiac hydatid cyst: A rare case report

**DOI:** 10.1097/MD.0000000000041811

**Published:** 2025-03-14

**Authors:** Muhammad Anas Kudsi, Abdoul Majid Sires, Jad Alhaq Wardeh, Ahmad Ismail, Tameem Azzawi

**Affiliations:** a Faculty of Medicine, University of Aleppo, Aleppo, Syria; b Department of General Surgery, Aleppo University Hospital, Aleppo, Syria; c Department of Cardiothoracic Surgery, Aleppo University Hospital, Aleppo, Syria.

**Keywords:** albendazole, cardiac cyst, echinococcosis, hydatid disease, parasitic infection

## Abstract

**Rationale:**

Hydatidosis, a larval cestode zoonotic infection caused by Echinococcus granulosus, predominantly affects the liver and lungs. While the disease is well-documented in these common sites, cardiac involvement remains exceedingly rare, with an incidence ranging from 0.02% to 2%. Among the cases of cardiac hydatidosis, cysts located at the apex of the heart are particularly uncommon, accounting for only 5.2% of reported instances.

**Patient concerns:**

A 43-year-old woman presented to the emergency department with fatigue and dyspnea on exertion. Physical examination was unremarkable, and laboratory tests showed normal hematology and coagulation test results, but positive indirect hemagglutination test for hydatid cyst raised suspicion of infection.

**Diagnoses:**

Cardiac ultrasound revealed an echo-lucent structure in the left ventricle, consistent with a cystic lesion. Further investigation with computed tomography (CT) scans identified a large 9 cm cardiac cyst at the apex of the left ventricle, a 3 cm cyst in the right lung, and multiple well-circumscribed cystic lesions in the right lobe of the liver. These findings strongly suggested the diagnosis hydatid disease.

**Interventions:**

The patient underwent surgery to evacuate the apical cardiac cyst liquid and remove its laminated layer. Albendazole was prescribed to prevent recurrence of the cyst and treat smaller cysts located in the liver and right lung.

**Outcomes:**

The patient recovered well with no evidence of cardiac abnormalities or recurrence during follow-up.

**Lessons:**

Cardiac hydatid disease, though rare, poses serious risks in endemic regions. Echocardiography and CT scans help in diagnosing hydatid cysts, measuring their size, and assessing their location. Surgical intervention is recommended, even in asymptomatic patients, to prevent cyst rupture and potential complications.

## 1. Introduction

Hydatid disease, or human cystic echinococcosis (CE), is an endemic tissue infection found in numerous places around the world that raise sheep, including the Mediterranean region, the Middle East, Australia, and South America.^[1]^ CE is a parasitic infection that mostly affects the liver and lungs. It is caused by the larval stage of the tapeworm Echinococcus granulosus. Approximately 80% of cases involve only 1 organ, most commonly the liver (73.4% of cases) and the lungs (19.6% of cases), though almost any region of the body might be impacted. Cardiac hydatid cysts are rare and occur in 0.02% to 0.2% of cases. The cardiac apex is an unusual location, occurring in just 5.2% of cardiac hydatid cyst cases. The prevalence is higher in young males (20–25 years).^[2]^ The main symptoms include cough, palpitations, dyspnea, and chest pain.^[3]^ Ultrasonography, computed tomography (CT), and magnetic resonance imaging are the main diagnostic modalities utilized to diagnose CE; serology is employed as an additional confirmatory diagnostic technique.^[2]^ Dysontogenetic cysts and cardiac myxomas are 2 differential diagnoses for cardiac hydatid cysts. Although it can be challenging at times, it is possible to differentiate cardiac echinococcosis by combining imaging techniques with the patient’s medical history and test result analysis.^[4]^ Surgical excision is the preferred course of treatment, even for asymptomatic cardiac hydatid cysts, as it results in a full recovery and a favorable prognosis.^[5]^ Potential hydatid cyst rupture into the pericardial cavity could end in cardiac tamponade, pericarditis, and pericardial effusion.^[6]^

## 2. Case presentation

**Figure 1.**
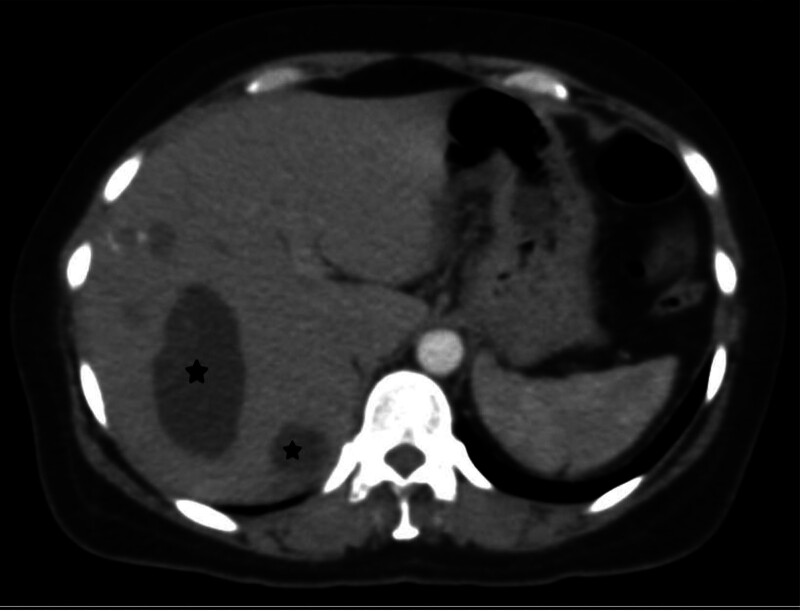
An axial contrast-enhanced computed tomography (CT) scan demonstrates the presence of hepatic hydatid cysts located in the right lobe of the liver (black stars). CT = computed tomography.

**Figure 2.**
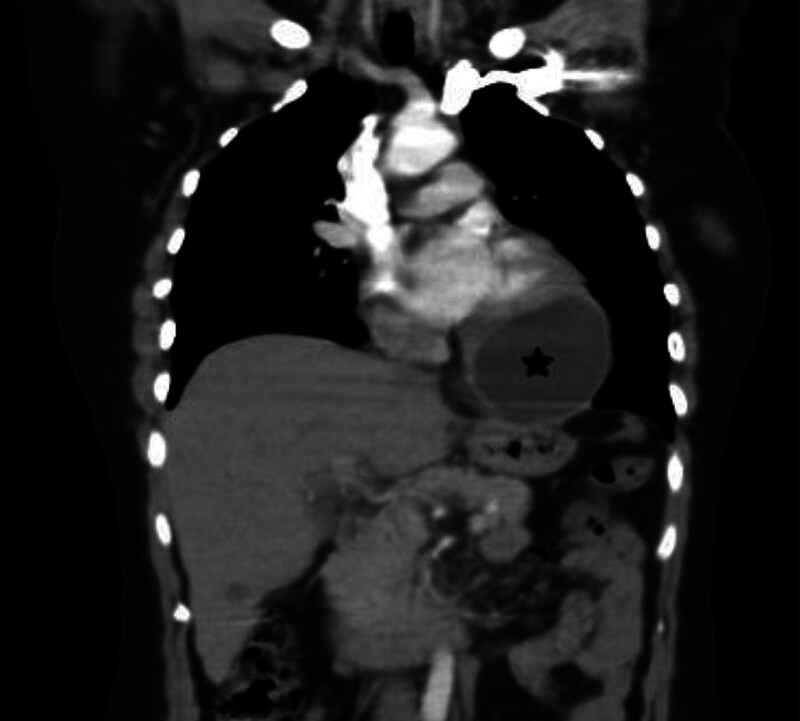
Coronal contrast-enhanced CT scan shows the cardiac hydatid cyst at the left ventricular apex (black star). CT = computed tomography.

**Figure 3.**
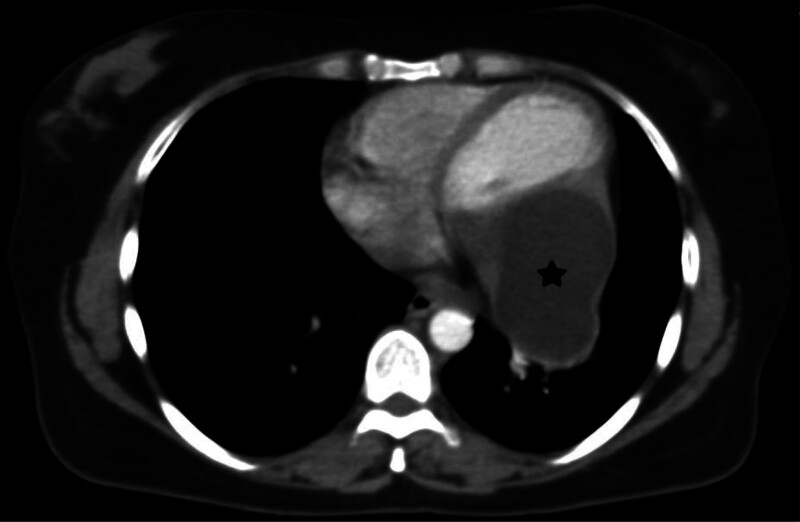
Axial contrast-enhanced CT scan shows the cardiac hydatid cyst at the left ventricular apex (black star). CT = computed tomography.

A 43-year-old woman presented to the emergency department with fatigue and dyspnea on exertion. Physical examination was unremarkable, and laboratory tests showed normal hematology and coagulation test results, but a positive indirect hemagglutination test (IHT) for hydatid cyst raised suspicion of infection. Cardiac evaluation revealed paroxysmal sinus tachycardia on echocardiography (ECG) and an echo-lucent structure in the left ventricle consistent with a cystic lesion on cardiac ultrasound. Abdominal CT demonstrated multiple well-circumscribed cystic lesions in the right lobe of the liver, with the largest measuring 4 cm with homogeneous hypoattenuation and mild contrast enhancement after intravenous administration of contrast material (Fig. 1). Chest CT identified a large 9 cm cardiac cyst at the apex of the left ventricle (Figs. 2 and 3) and a 3 cm cyst in the right lung, suggesting potential hydatid disease due to the cystic nature of lesions across multiple organ systems, necessitating a multidisciplinary approach for management, including monitoring and possible surgical intervention for the cardiac cyst.

**Figure 4.**
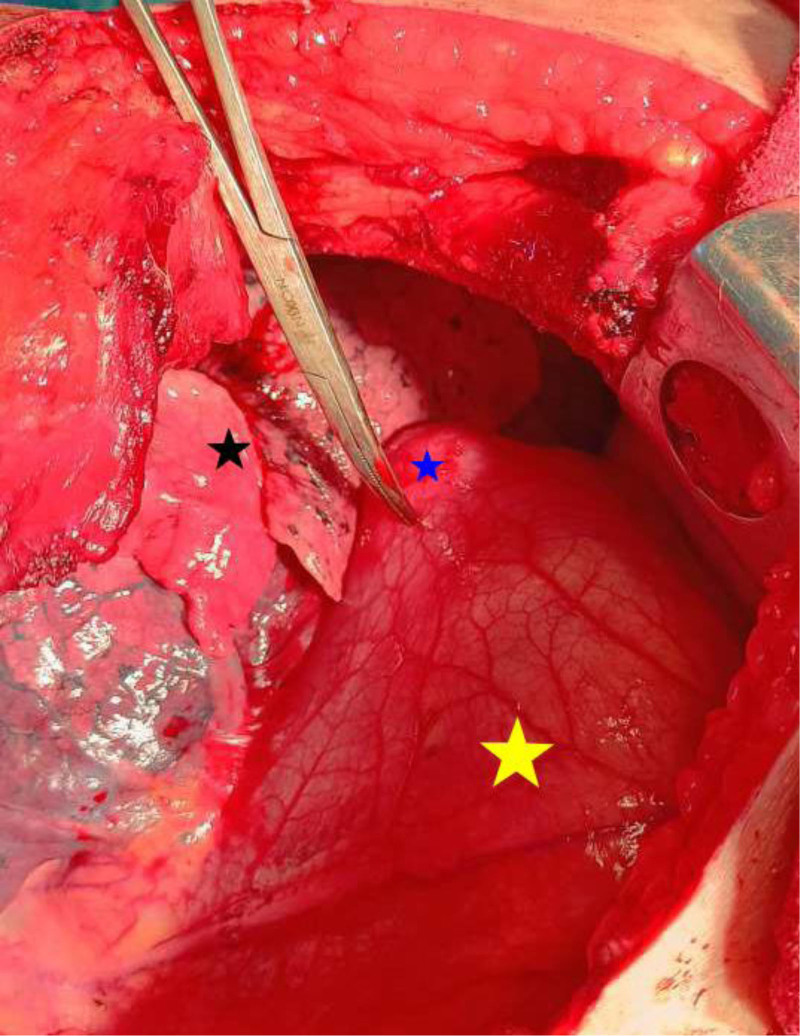
Intraoperative image illustrating the superficial cardiac hydatid cyst located at the apex of the heart (blue star), alongside the surrounding pericardium (yellow star) and adjacent lung tissue (black star) prior to cyst resection.

The patient was admitted to the hospital for symptoms management and was started on weight-based albendazole (0.4 g twice a day) for 3 months before surgery. The patient improved clinically and was taken to the operating room for left anterolateral thoracotomy, followed by an incision of the pericardium while carefully avoiding major vascular structures including pericardiophrenic vessels. Pericardial adhesiolysis was performed to detach the pericardium from the external wall of the cyst located within the left ventricular apex myocardium (Fig. 4). The cyst was accessed for aspiration to evacuate its contents, after which a hypertonic saline solution was instilled. The adventitia of the cyst was then incised to facilitate the removal of the laminated layer, and a thorough inspection of the cyst margins revealed reassuring thickness in the cyst cavity. Subsequently, the cystic cavity was closed, and the pericardium was sutured with a retained window for drainage. Notably, the entire procedure was conducted with a beating-heart (off-pump) technique resection since the cyst was superficial and unrelated to the cardiac chambers (see Video S1, Supplemental Digital Content, http://links.lww.com/MD/O496 which demonstrates the cardiac hydatid cyst surgical resection).

Supplemental medical therapy with albendazole (0.4 g twice a day) for 6 months was prescribed to reduce the risk of recurrence and treat smaller cysts located in the liver and the right lung. The postoperative period was uneventful and the patient was discharged from the hospital within 5 days postoperatively. During the follow-up, no evidence of cardiac abnormalities or recurrences was detected during repeat CT scans and echocardiography.

## 3. Discussion

Hydatidosis is a larval cestode zoonotic infection transmitted by Echinococcus granulosus. This disease causes cysts called hydatid cyst in the internal organs of intermediate hosts including humans. The definitive hosts are carnivores like dogs, and infection occurs when humans consume eggs or gravid proglottids which pass out in the feces of these hosts.^[7]^ Hydatidosis frequently occurs in Southern and Eastern Europe and the Middle East, where dogs are often fed sheep and cattle innards, leading to high levels of E. granulosus infestation in dogs.^[4]^

In about 80% of hydatid cyst cases, only a single organ is affected, mainly the liver (73.4% of cases) and the lungs (19.6% of cases).^[2]^ However, any body part can be affected, that includes the heart which is an uncommon site for hydatid cyst, with an incidence of 0.02% to 2%. It may occur due to hematogenous spread or rupture of hydatid cysts in the lungs.^[8]^ Cardiac hydatid cysts most frequently affect the left ventricle (34%), followed by the interventricular septum (20.8%), right ventricle (16.2%), pericardium (11.7%), right atrium (7.3%), apex (5.2%), and left atrium (4.9%).^[8]^ Our case, involving a hydatid cyst in the apex, stands out as a rare occurrence given the typical distribution of these cysts within the heart.

Cardiac hydatid cysts can affect people of all ages in areas where the disease is prevalent. However, the presence of nonspecific symptoms and changes in the ECG make the diagnosis more challenging,^[1]^ as seen in our case. Hydatid cysts typically remain asymptomatic until they grow in size. Once they expand, patients may experience a range of symptoms including heart failure, allergic reactions, arrhythmias, exertional angina, cardiac tamponade, heart block, and even sudden death.^[9]^ While our patient exhibited paroxysmal sinus tachycardia on her ECG, normal sinus rhythm is the most common ECG finding observed in patients with cardiac hydatid cysts.^[1]^ Serology is helpful in diagnosing echinococcosis. Initial screening often involves indirect IHT and enzyme-linked immunosorbent assay tests to detect IgG, IgM, or IgE antibodies. The IHT test is positive in more than 80% of liver hydatid cysts.^[9]^ Cardiac hydatid cysts can be diagnosed using various imaging techniques, including transthoracic echocardiography, CT, and magnetic resonance imaging. While transthoracic echocardiography provides valuable information, its ability to show the cyst’s relationship with surrounding structures may be limited. CT is the most effective method for revealing calcification in the cyst wall; however, it may be insufficient for clearly visualizing the heart chambers due to motion artifacts caused by the heart’s movement.^[8]^

Surgical intervention is the preferred approach for cardiac hydatid disease, even in asymptomatic patients, due to the potential risk of cyst rupture leading to anaphylaxis and metastatic complications. To reduce the likelihood of recurrence, antihydatid medications are crucial as an adjunct to surgery.^[10]^

While superficial cysts can be successfully removed with a beating-heart technique (like our case), resection using cardiopulmonary bypass has been recognized as the safest method since 1962. This approach minimizes the risk of cyst contents leaking during surgery.^[5]^ However, ventricular myocardial hydatid cysts unrelated to the cardiac chambers can be surgically treated without the need for cardiopulmonary bypass, as described by Birincioğlu et al.^[11]^

Given the potential for life-threatening complications associated with cardiac hydatid cysts, it is crucial to screen for them in individuals diagnosed with liver or lung echinococcosis.^[4]^

## 4. Conclusion

While uncommon, cardiac hydatid disease is a serious medical concern that should be considered when evaluating patients with cardiac or respiratory symptoms in regions where the disease is prevalent. Initial assessments should include tests to rule out this condition. Echocardiography and CT scans are valuable tools for accurately identifying hydatid cysts, measuring their size, and understanding their relationship to nearby tissues.

## Author contributions

**Conceptualization:** Muhammad Anas Kudsi, Abdoul Majid Sires, Tameem Azzawi.

**Investigation:** Abdoul Majid Sires, Ahmad Ismail, Tameem Azzawi.

**Methodology:** Ahmad Ismail, Tameem Azzawi.

**Project administration:** Tameem Azzawi.

**Software:** Ahmad Ismail.

**Supervision:** Abdoul Majid Sires, Ahmad Ismail, Tameem Azzawi.

**Validation:** Muhammad Anas Kudsi, Abdoul Majid Sires, Jad Alhaq Wardeh, Tameem Azzawi.

**Visualization:** Muhammad Anas Kudsi, Abdoul Majid Sires, Jad Alhaq Wardeh, Tameem Azzawi.

**Writing – original draft:** Muhammad Anas Kudsi, Abdoul Majid Sires, Jad Alhaq Wardeh.

**Writing – review & editing:** Muhammad Anas Kudsi, Abdoul Majid Sires, Jad Alhaq Wardeh.

## Supplementary Material


